# Role of Pseudoexons and Pseudointrons in Human Cancer

**DOI:** 10.1155/2013/810572

**Published:** 2013-09-24

**Authors:** Maurizio Romano, Emanuele Buratti, Diana Baralle

**Affiliations:** ^1^Department of Life Sciences, University of Trieste, Via A. Valerio 28, 34127 Trieste, Italy; ^2^International Centre for Genetic Engineering and Biotechnology (ICGEB), 34149 Trieste, Italy; ^3^Human Development and Health, University of Southampton, Duthie Building, Mailpoint 808, Southampton General Hospital, Tremona Road, Southampton SO16 6YD, UK; ^4^Human Genetics Division, University of Southampton, Duthie Building, Mailpoint 808, Southampton General Hospital, Tremona Road, Southampton SO16 6YD, UK

## Abstract

In all eukaryotic organisms, pre-mRNA splicing and alternative splicing processes play an essential role in regulating the flow of information required to drive complex developmental and metabolic pathways. As a result, eukaryotic cells have developed a very efficient macromolecular machinery, called the spliceosome, to correctly recognize the pre-mRNA sequences that need to be inserted in a mature mRNA (exons) from those that should be removed (introns). In healthy individuals, alternative and constitutive splicing processes function with a high degree of precision and fidelity in order to ensure the correct working of this machinery. In recent years, however, medical research has shown that alterations at the splicing level play an increasingly important role in many human hereditary diseases, neurodegenerative processes, and especially in cancer origin and progression. In this minireview, we will focus on several genes whose association with cancer has been well established in previous studies, such as *ATM*, *BRCA1/A2*, and *NF1*. In particular, our objective will be to provide an overview of the known mechanisms underlying activation/repression of pseudoexons and pseudointrons; the possible utilization of these events as biomarkers of tumor staging/grading; and finally, the treatment options for reversing pathologic splicing events.

## 1. Introduction

Starting from the first description of alternative splicing and constitutive splicing processes in 1977 [1–3], the importance of this process that guarantees the correct flow of information from transcription to translation in eukaryotic cells has continued to grow exponentially.

In particular, one major branch of research in this area has the aim to investigate and characterize the cellular macromolecular machine (i.e., the spliceosome) that is physically responsible for the cutting and joining of intronsexons by catalyzing two transesterification reactions [4, 5] and the mechanisms that ensure its fidelity [6]. As a result, research in spliceosome composition and functioning has been complemented by studies aiming to understand the sequences and molecules that determine under which conditions a particular exon or intron is selectively recognized and included in the mature transcript. Therefore, after the basic elements that define introns and exons and are composed by donor and acceptor splice sites plus the branch point sequence, there was the discovery of enhancer and silencer elements that can affect either positively or negatively the way these basic elements are recognized by the spliceosome [7–9]. Usually, enhancer and silencer elements are bound by members of the SR and hnRNP protein families, respectively.

The recruitment of these proteins to specific sites is crucial for their activity as depending on their position with respect to the basic elements their roles can be antagonistic or, in some case, agonistic [10, 11]. Global analyses of the ways these factors cooperate and influence each other in shaping the splicing process has only recently begun to shed light on how they promote or hinder exon recognition in a co-ordinated manner [12–14].

Rather unexpectedly, these studies have shown that SR proteins interact not only with alternatively spliced exons but also with constitutively spliced exons, and that their major role consists in recruiting the splicing machinery to splice sites [15, 16].

On the other hand, hnRNP proteins bind to nascent pre-mRNA and influence splicing decision through a complex and finely tuned network of protein/protein interactions. The mechanisms of hnRNPs actions on splicing is generally less defined than those of SR proteins [17].

In addition to these elements, we now also know that splicing choices are affected by a myriad of other factors, ranging from chromatin modifications [18, 19], transcriptional factors [20], RNA secondary structure [21], short noncoding RNAs [22, 23], and various cellular stresses [24].

In parallel to these mechanistic studies, another branch of splicing research has also addressed the functional importance of alternative splicing processes in biological pathways and in particular the way that alternative splicing isoforms of proteins can acquire different or even antagonistic biological properties [25, 26]. Because of this ability to expand the proteome of cells, alternative splicing has represented a very useful and powerful tool that allows cells to execute the various expression programs which underlie many fundamental needs of higher organisms: from general needs such as controlling normal development and tissue-specific expression of proteins, to highly specialized processes such as DNA damage response or microRNA biogenesis [27–33]. Moreover, the rearrangements that a pre-mRNA undergoes during the splicing process are also advantageous in terms of providing a longer half-life and better translational capacity, something that has recently begun to be exploited by the biotechnology industry [34].

Considering all these necessities and advantages, it is therefore not surprising that the number of genes that are subject to alternative splicing in eukaryotic organisms has been steadily growing. Indeed, it was recently estimated that more than 90% of mammalian protein coding genes can produce at least one splicing-derived isoform expressed at potentially significant biological levels [35]. 

The complexity of this system, however, also puts spliceosome functioning at risk of being impaired by the occurrence of single-point mutations in splicing regulatory elements, deletions/insertions, genomic rearrangements, and alterations at the splicing factor expression level [36]. Any of these alterations can result in a variety of aberrant splicing outcomes that usually include aberrant exon skipping, cryptic splice site selection, intron retention, and pseudoexon activation [37, 38]. As expected, many of these changes can lead directly to the occurrence of disease in humans, and it has now been estimated that a sizable proportion of all gene mutations leading to disease can be directly connected with the presence of a splicing defect [7, 39].

Importantly, the introduction of novel technologies that allow fast profiling of the transcriptome seems promising for simplifying investigation of which genomic variability and plasticity events allow cancer cells to tailor specific functional units from the available exons of a gene. For example, recent RNA sequencing of the breast cancer transcriptome has revealed many new splicing alterations that were not previously described. In particular, for example, it was reported that 423 primary transcripts (derived from 377 genes) were differentially spliced in triple-negative breast cancer samples (generating 496 novel isoforms) [40]. By analyzing non-triple-negative breast cancer samples, the same study found that 270 and 460 primary transcripts (derived from 242 and 387 differentially spliced genes, resp.) generated 331 and 550 novel isoforms that were not present in normal breast tissue [40].

## 2. Aberrant Splicing Events in Cancer Genes

It has become clear that in several human pathologies, including cancer, the alternative splicing profile is aberrantly modified in a specific manner and in ways that can favor the growth and survival of cancer cells [41–44]. The generation of these aberrant splicing profiles can occur in many ways, such as through the re-expression of developmentally regulated isoforms that had previously been shut off following early developmental stages [45, 46], by affecting the splicing profiles of genes that are implicated in tumor progression [47, 48], or through other mechanisms that generally have anti-apoptotic/metastatic consequences and that can affect response to therapies [49, 50].

In addition, several studies have defined that cancer-associated splicing alterations arise not only from mutations in the cancer-related genes but derive also from variations in the expression and/or activity of splicing regulatory factors [51]. For example, it has been well established that the levels of SR- and hnRNP-proteins undergo changes associated with transformation and progression of cancers [44, 52]. Strikingly, it has been recently reported that some members of the SR protein family of splicing factors and other components of the spliceosomal cellular machinery can actually act as oncoproteins and play a direct role in promoting tumor origin and progression [53–55] and that external stimuli such as hypoxia can cause the aberrant redistribution of important splicing factors such as Tra2 and promote the expression of tumor-promoting splicing isoforms [56].

The case of *Bcl-X* (BCL2L1) gene, belonging to the BclII family and implicated in the control of mitochondrial breakdown during apoptosis, is emblematic of this concept. For example, two splicing Bcl-X isoforms can arise from the use of two alternative 5′ splice sites within exon 2 and lead to the synthesis of a short apoptosis-promoting protein (Bcl-XS) and to a long antiapoptotic form (Bcl-XL) [57]. Different splicing factors, including Sam68, hnRNPA1, SF2/ASF, hnRNP F/H, hnRNP K, SAP155, and SRp30c have been found to be involved in the selection of the two competing alternative 5′ splice sites that give rise to these two isoforms [58–61]. In addition, recent studies have shown that the elongation and splicing-related factor TCERG1 can bind to the Bcl- X pre-mRNA and promote the proapoptotic Bcl-XS 5′ splice site in a promoter-dependent manner [62].

Finally, the production of the proapoptotic Bcl-XS splice variant seems to be improved by the core (Y14 and eIF4A3) and auxiliary (RNPS1, Acinus, and SAP18) components of the exon junction complex (EJC) [63], suggesting that EJC-associated components can regulate apoptosis at the alternative splicing level and represent a further level of vulnerability of cancer.

Therefore, one of the most interesting research areas in this field consists in the identification of cancer-specific splice variants or the aberrant expression of splicing-affecting proteins that could lead to their generation. Examples of both these events, in fact, have already been shown to occur in some individual types of cancer, such as breast and ovarian cancer [64, 65]. Another interesting research area in aberrant splicing events connected with tumors is the occurrence of particular types of splicing defects that involve the inclusion of “new” sequences (known as pseudoexons) in the mature mRNA of cancer-related genes. Rather more rarely, the opposite has also been shown to occur: the aberrant recognition of intronic sequences (pseudointrons) within normal exons. In this review, we have also decided to provide particular attention to these events as they are probably more common than previously considered and have not yet been the subject of particular attention.

One of the reasons why these two events are particularly interesting is that these types of defects are ideally suited for novel therapeutic effector molecules that are based in RNA biology. In the case of pseudoexons and pseudointrons, in fact, the major advantage of targeting this type of inclusion events is that the antisense oligonucleotides would be targeted against normal intronic sequences and thus would not remain bound to the mature mRNA (possibly to interfere with later stages of RNA processing such as export/translation).

## 3. Pseudoexon Activation in Cancer

In the pre-mRNA splicing field, the term “pseudoexon” has been introduced to describe exonic-like sequences that are present within intronic regions but are ignored by the spliceosomal machinery. A closer look at these sequences has often provided a reason for their inability to be recognized as normal exons: the presence of intrinsic defects in their apparently viable donor and acceptor sites [66] or of silencer elements [67–69] and the formation of inhibiting RNA secondary structures [70–72].

From a functional point of view, in most cases of pseudoexon insertion, the presence of an extraneous exon within the mature mRNA causes either the disruption of the translational reading frame or the insertion of novel amino acid sequences following translation. As a result, the normal biological properties of the resulting protein are very likely disrupted, and this can be associated with the development of disease.

Unfortunately, it is still quite hard to identify reliable pseudoexon insertion events in human genes implicated in cancers by just performing a general interrogation of databases (matching 22719 ENSEMBL protein coding genes versus 31057 entries of CanGEM Gene list) [73]. In fact, at present, it is only possible to retrieve strong candidates for alternative splicing events in protein coding genes (Table 1). As expected, a similar situation was seen when pseudogenes were investigated (defined as genomic DNA sequences similar to normal genes but nonfunctional, although some can still be transcribed). In this case, inspection of 14775 pseudogenes returned a list of alternative splicing hits from which it is not easy to distinguish real events in expressed pseudogenes (Table 1).

More recently, some bioinformatic studies have tried to extrapolate the presence of pseudoexons in cancer tissues by developing new analysis methods of GeneChip gene expression array data [74, 75]. In this way, by comparing normal cerebellum and medulloblastoma tumors, it was possible to predict 811 significantly different expressed pseudoexons (derived from 577 genes). In addition, when nonmetastatic and metastatic medulloblastomas were compared, 13 pseudoexonic sequences were significantly expressed in a differential manner (derived from 8319 strong candidates). However, it should be noted that no experimental validation was carried out to support these predictions. Therefore, presently, manual annotations remain the most reliable system for identifying real pseudoexons.

Fortunately, the scientific community has recently identified a certain number of cases where pseudoexon inclusion has been validated in detail. For this reason, Table 2 reports all the cryptic exons described in the literature that are localized within genes whose expression was altered in cancer.

First of all, regarding the mechanisms underlying pseudoexon activation, it is interesting to note that these phenomena are caused by inherited mutations resulting in the insertion of intronic sequences in the mature mRNA [38]. In this respect, it is interesting to note that among the genes presenting pseudoexon “awakening” directly associated with cancer origin (Table 2), the creation of new splicing donor (12 events) and acceptor (7 events) sites represents the more frequent occurrence (48% and 28% of the listed events, resp.). Some of the most paradigmatic examples of cancer genes such as *BRCA1*, *BRCA2*, *NF1*, and *ATM* are included in these two sets.

On the other hand, among pseudoexons described in cancer-related genes (Table 3), the creation of 5′ splice sites and of 3′ splice sites comprise 55% and 15% of the listed records, respectively. Moreover, pseudoexon insertion can also be triggered by the deletion of nearby donor or acceptors splice sites (4 events in both lists), highlighting the importance of the genetic milieu in splicing decisions.

A deeper look into the mechanisms underlying pseudoexon activation has revealed the involvement of hnRNPs in regulation of these events. In particular, the partial inclusion of a pseudoexon of *NF1* gene has been found to arise from a novel intronic mutation c.31−279A>G intron 30, creating a new acceptor splice site and activation of a cryptic 5′ splice site [76]. It has been found that both PTB and nPTB play an active role in the pseudoexon splicing by repressing its inclusion [76]. This finding is particularly interesting since it further supports the hypothesis that PTB/nPTB might have a general role in repressing weak exons and in particular most of pseudoexons [77].

Another example of mutation causing pseudoexon activation consists in the creation of novel branch site (Table 2, one event). This latter case is associated with a peculiar mutation (IVS5 ds +232 G−A) that leads to pseudoexon inclusion in *NF2* gene by creating a consensus branch point. In particular, it has been found that the G>A transition, occurring at position −18 from the acceptor site of NF2 IVS5, works in combination with existing consensus splice acceptor and donor sites and causes the formation of an extra exon 5a in the *NF2* gene that introduces a premature stop codon [98].

Finally, in spite of being lowly represented, gross genomic rearrangements can bring together or expose splice site sequences that would normally be very distant from each other or absent form the original sequence (one event).

**Figure 1:**
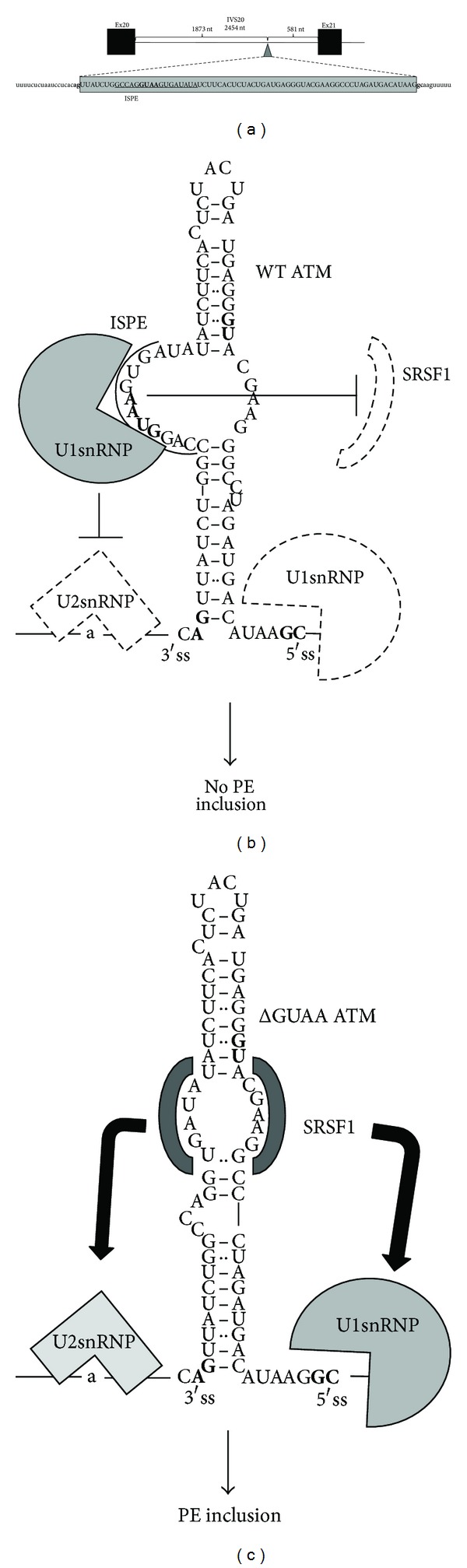
ATM pseudoexon activated in cancer. (a) Scheme of the ATM pseudoexon insertion caused by a 4 nt deletion (GUAA) occurring 1873 nt downstream from the donor splice site of ATM exon 20. The solid lines indicate introns. Dotted lines include the 65 nt-ATM pseudoexon (gray box). The intron-splicing processing element (ISPE) complementary to U1 snRNA, the RNA component of the U1 small nuclear ribonucleoprotein (snRNP), is underlined. (b) Schematic model of U1snRNP mediated inhibition of pseudoexon insertion in normal conditions. In this model, U1snRNP binding to the ISPE blocks pseudoexon inclusion by inhibiting recruitment of U2snRNP to the 3′splice site region and SRSF1 binding to the stem loop of the pseudoexon (shown as dotted lines). (c) In the case of the disease associated ATM ΔGUAA deletion, the internal U1snRNP binding site is no longer present due to the deletion of the ISPE. This leads to a more efficient binding of SRSF1 to the enhancer site and stabilization of the U2snRNP interaction with the branch site and U1snRNP with the 5′gc donor site.

Pseudoexon activation can also be caused by mutations that cause the creation/deletion of splicing regulatory elements (SREs, 6 events). Among these events, one of the most representative examples of the complex regulatory networks that can underlie pseudoexon insertion is represented by the identification of an unusual splicing defect directly related with neoplasia (Figure 1).

In the case of this particular event, a patient affected by ataxia-telangiectasia was found to carry a 4 nt-deletion (GTAA) within intron 20 of *ATM* gene [80]. This deletion, termed intron-splicing processing element (ISPE), caused the inclusion of a 65 nucleotide “cryptic exon” in the ATM mRNA (Figure 1(a)). The mechanism through which this occurs has been well characterized in recent studies.

In normal conditions, the repression of the 3′ss in the wild-type pseudoexon sequence (ATM WT) was shown to be a direct consequence of U1snRNP binding in correspondence to the ISPE sequence. The importance of this U1snRNP binding was twofold: first of all, to sterically hinder 3′ss recognition by U2snRNP and secondly, to also inhibit binding of the SRSF1 splice factor to the stem-loop sequence formed by this pseudoexon (Figure 1(b)) [71, 72]. As a result, this internal U1snRNP binding event to the ISPE sequence causes an unproductive U2snRNP association with the 3′ss that results in the complete inhibition of pseudoexon inclusion in normal conditions (Figure 1(b)). Following the deletion of the GUAA motif observed in the patient, U1snRNP binding to the ISPE is relieved, and SRSF1 is also free to bind to the internal enhancer site that becomes available (Figure 1(c)). Taken together, these two events result in efficient recruitment of U2snRNP to the branchsite and a better recruitment of U1snRNP to the rather inefficient 5′gc splice site that lead to efficient inclusion of the pseudoexon in the mature ATM mRNA [72].

This example clearly shows the complexity of pseudoexon activation events that relies on a variety of factors well beyond the simple initial mutation (i.e., 5′ss or 3′ss creation), and that can include the eventual presence of enhancer and silencer elements within the cryptic exon or intro, RNA secondary structures, and probably several other mechanisms that have not yet been described in detail.

## 4. Pseudointron Activation in Cancer

Pseudointrons (PSIs) represent an intriguing set of intron-like sequences localized within exons that can undergo alternative splicing and that therefore can be included in or excluded from the ORF within the final mRNA species.

Although the examples of pseudointrons reported in the review are not associated with activating mutations, we have included them because of the peculiarity of events insofar that they do not represent simple intron retention events, but rather alternatively spliced intraexonic sequences whose behaviour can resemble that of introns under particular circumstances (e.g., occurrence of previous splicing events in the processed transcript). It is this regulated use of the intraexonic splice sites that fits well with the idea that these intraexonic splice site represent “false” introns, even in the absence of genetic alterations.

In general, however, pseudointrons are less well described compared withpseudoexons probably because of the difficulty in detecting them using available technology (a situation that may well soon change for the better because of the introduction of more sensitive and comprehensive technical approaches such as RNA sequencing). At the moment, only three examples of PSI have been characterized, and for at least two of these pseudointrons there are data supporting their relevance in the pathogenesis of cancer.

### 4.1. Fibronectin: IIICS-C5

Fibronectin (FN) is an extracellular matrix protein whose functions range from cell adhesion and migration to wound healing and oncogenic transformation [145]. The functional complexity of FN is related to the structural diversity arising from cell type-specific alternative splicing [146]. The fibronectin pre-mRNA undergoes alternative splicing primarily at three sites: two extra domain exons encoding extra structural repeats and a region of nonhomologous sequence called the type-III connecting segment (IIICS).

The IIICS domain is divided into three subdomains of 75 bp, 192 bp, and 93 bp, respectively, that can be alternatively spliced, thus generating up to fivevariant mRNAs in humans [147, 148]. The subdomains of 75 bp and 93 bp encode for two cell-specific binding sites, CS1 (residues 1–25 of the IIICS), and CS5 (residues 90–109 of the IIICS), respectively, that interact with the integrin *α*4*β*1 with different affinity [149, 150]. Indeed, the CS1 site has approximately 20-fold higher affinity for integrins than the CS5 site [151].

**Figure 2:**
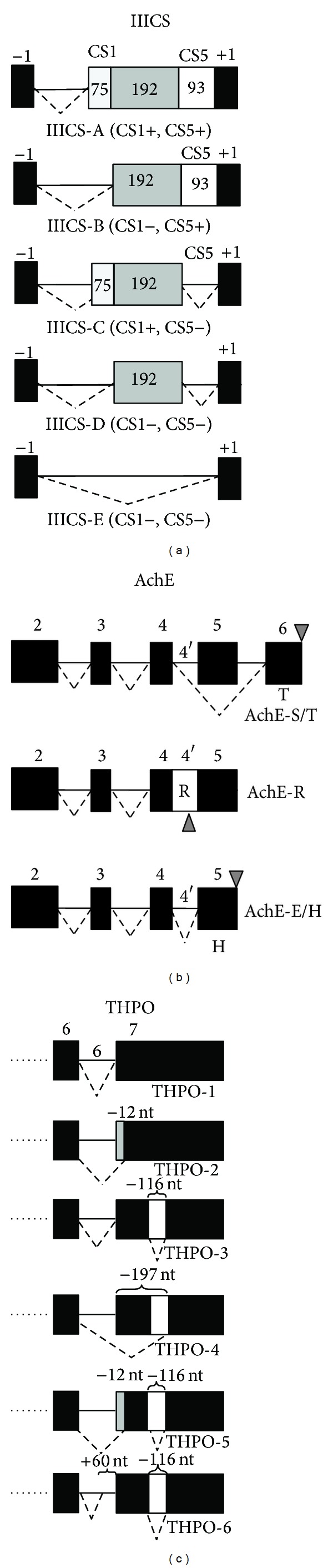
Pseudointrons whose alternative splicing can be altered in tumors. Schemes of pseudointron alternative splicing events shown to occur within the Fibronectin (IIICS, (a)), Acetylcholinesterase (AchE, (b)), and Thrombopoietin (THPO, (c)) genes. Pseudointrons are shown as white boxes in IIICS (CS5-93 nt), AchE (4′-R), and THPO (116 nt). Solid lines indicate introns and dotted lines distinguish the different splicing events. The C-terminal sequence of each splicing variant for each gene is also shown. For IIICS and THPO, additional exonic elements undergoing alternative splicing are shown as gray boxes. In the *AchE* gene, gray triangles indicate stop codon within different ORFs. See text for definition of each splice variant.

Because of its intraexonic localization and splicing behavior, the 93 bp segment can be considered a pseudointron (Figure 2(a)). Different tissue-specific and disease-associated changes in the variants of the IIICS region have been reported [152–154], and the CS5 pseudointron is upregulated in foetal tissue and in adult liver [155].

For the purpose of this review, it is important to note that alternative splicing of the IIICS-CS5 subdomain can influence the onset and progression of cancers by affecting Fibronectin activities related to tissue organization and cell-ECM interactions. In keeping with this hypothesis, *in vitro* studies have shown that CS5 can modulate the spreading of melanoma cells [151].

### 4.2. Acetylcholinesterase: AChE-R

Acetylcholinesterase is a serine specific hydrolase that hydrolyzes acetylcholine, and its main function is related to the clearance of the neurotransmitter from the synaptic cleft [156]. Nonetheless, the observation that the expression of AChE is not limited to cholinergic tissues has suggested the presence of additional functions [157].

From the splicing process point of view, mammal AChE has been shown to undergo alternative splicing resulting in expression of 3 isoforms, called R (“readthrough”), H (“hydrophobic”), and T/S (“tailed” or “synaptic”), that differ in their subcellular localization, tissue distribution, and developmental pattern of expression [158]. Whereas, AChE-H or AChE-T is the most abundant and common isoforms, the AChE-R expression is low and restricted in specific cell lines, cell differentiation stage, or muscle development [159, 160].

Interestingly, the AChE-R mRNA arises by retention of pseudointron4, that leads to generation of the E1-E2-E3-E4-I4-E5 mRNA transcript (Figure 2(b)). Since pseudointron 4 includes a stop codon when it is included in mature RNA, the resulting protein will be prematurely terminated by a C-terminus translated from the 5′ region of I4. This variant is expected to remain monomeric [161].

In general, the AChE-R transcript can be coexpressed with the AChE-T or AChE-H transcripts, and variations in their relative levels seem to be important for modulation of AChE functions, as well as for the pathogenesis of neurological and autoimmune disorders [161, 162]. Intriguingly, recent studies have found that AChE-R mRNA accumulates in primary human astrocytomas, and that its presence is correlated with their grade of aggressiveness [163]. In human U87MG glioblastoma cells, it was found that AChE-R protein variant can support proliferation of glioblastoma tumors by forming a complex with the scaffold protein RACK1 and protein kinase Ce [164].

In addition, increased levels of an N-terminally extended N-AChE-R isoform were detected in human testicular tumors indicating that the generation of the AChE-R variant can be associated with the utilization of an alternative promoter in the transformed cells.

Finally, related functional studies have suggested that both AChE-R variants (AChE-R and N-AChE-R) might be crucial for increasing the cellular ATP levels and might support selective metabolic advantages as well as genotoxic resistance by altering *p73* gene expression [165]. Although it is not yet clear whether the AChE-R (as well as the other AChE splicing variants) can be related directly to tumorigenesis, the observed downregulation of all these splicing isoforms in colorectal carcinoma suggests a crucial role for tumor development [166].

### 4.3. Thrombopoietin: THPO-3,THPO-5, and THPO-6

A third example of pseudointron possibly implicated in cancer pathogenesis is found within the Thrombopoietin (*THPO*) gene. THPO is the most important cytokine for regulation of platelet production, and its expression has also been reported in skeletal muscle, ovary, testis, and central nervous system (CNS) [167–169].

The *THPO* gene shows a complex pattern of alternative splicing [170]; among the six splice variants that can be generated, three of them (THPO-3, THPO-5, and THPO-6) show the peculiar alternative splicing of 116 nucleotides within exon 6 (that can therefore be classified as a pseudointron) (Figure 2(c)) [171].

Contrary to the Fibronectin IIICS-C5 and AchE-R pseudointrons, the involvement in cancerogenesis of the variants deriving from the alternative splicing of the 116 nt pseudointron is less characterized. Nonetheless, functional studies have demonstrated that the protein translated from mouse THPO-3 is not secreted efficiently [172–174]. These results have suggested that THPO-3 might be implicated in the fine regulation of THPO full-length levels and in the modulation of several biological functions beyond thrombopoiesis. Interestingly, parallel studies have also shown an altered expression pattern of the THPO-3, THPO-5, and THPO-6 splicing variants in human carcinomas [175]. As a result, these observations have led researchers to hypothesize that alterations in alternative splicing of the 116 nt-THPO pseudointron might be employed as biomarker of tumor formation or progression [170].

In conclusion, although currently there are few examples of pseudointron removal, at least three of these events have been associated with different levels of cancerogenesis, ranging from transformation to cancer progression. Therefore, it is advisable that future high throughput screening analysis should be able to better associate the alternative splicing process of these pseudointrons with tumoral events. In particular, with regard to their use as possible biomarkers of transformation or of cancer staging/grading.

## 5. Therapeutic Outlooks

Regarding therapies, it is likely that all this information on the importance of alternative splicing for tumor development and progression may soon become extremely important for the development of novel therapeutic effectors in the fight against cancer [176, 177]. Indeed, one of the reasons why these defects are particularly interesting is the opportunity they provide for RNA therapies and the chance to actually make the jump from the bench to the bedside.

Currently, the field of RNA therapy is rapidly growing especially in the inhibition of undesirable splice site choices by the use of antisense oligos [178–180]. This technique, pioneered by Dominski and Kole [181], is based on the use of suitably modified antisense oligonucleotides that target specific sequences within introns and exons and block the recognition by their cellular binding factors. Therefore, they are ideally suited to block unwanted splice sites that may become activated following their creation or activation. It is interesting that the role of using antisense strategies to regulate exon inclusion also occurs in physiological conditions. An example of this is snoRNA HBII-52 in the regulation of exon Vb inclusion in the serotonin receptor 2C [182].

To this moment, a few pilot studies aimed at inhibiting the inclusion of a pseudoexon in patient cell lines have already been reported. In particular, antisense oligonucleotides have been used to target newly created 5′ or 3′ splice sites deep within intronic regions of the *NF1* gene to restore the normal splicing profile [91, 92]. Similar approaches have also been successfully used to target deep intronic mutations causing pseudoexon activation within BRCA2 intron 12 (c.6937+594T>G) [88], ATM intron 11 (c.1236−405C>T) [82], and ATM IVS19 (c.2639−384A>G) [79].

Recent advances in antisense-mediated exon skipping for DMD suggest that not only the splice sites but also exonic splicing enhancer sites or branch points might represent potential targets for inducing pseudoexon skipping [183, 184], and that the action of ASOs might be potentiated by coadministration of drugs, as shown for Duchenne muscular dystrophy [185].

An intriguing variation on the theme of the antisense oligonucleotides, successfully tested for inhibiting the 5′ splice site of Bcl-XL, might consist in the use of tailed oligonucleotides containing a portion annealing to sequences immediately upstream of the target donor splice site joined to a nonhybridizing 5′ tail that includes binding sites for the hnRNP A1/A2 proteins [186].

In spite of being prominent, antisense technologies are not the only possibility available for targeting these aberrant splicing events. An alternative to antisense usage is the development of siRNA molecules capable of targeting the newly created system. In this manner, only the mRNAs containing the pseudoexon might be selectively degraded leaving any residual normal mRNA to be properly processed [187]. The disadvantage of simply degrading the pseudoexon-bearing transcript (rather than acting on the splicing event itself) is that everything depends on the level of pseudoexon inclusion (less than 100%) and on the status of the other allele (i.e., whether it is normally expressed or not).

Finally, small molecule compounds might be also considered as possible therapeutic options in order to prevent the recognition of pseudoexons or modulate the recognition of pseudointrons. For example, it has been shown that sodium butyrate, an histone deacetylase inhibitor known to upregulate the expression of Htra2-beta1 and SC35, promotes skipping of the pseudoexon activated by the CFTR 3849+10kbC>T mutation and can restore functional CFTR channels [188]. In addition, the splicing of different NF1 skipped exons as a result of mutations in cis-acting sequences has been restored with administration of the small molecule kinetin [189].

More recently, a growing body of research indicates that TALE nucleases (TALENs) have been used with great success in a number of organisms to generate site-specific DNA variations [190]. As a result, this approach is another good candidate for an innovative therapeutic anticancer strategy for correction of deep intronic mutations that create novel splice sites.

Of course, crossing the difficult gap between the bench to the bedside will not be immediate even in the presence of highly efficient therapeutic molecules, whether antisense oligos, siRNAs, or others. Indeed, before any of these can be used in humans to treat cancer, several additional factors need to be considered. First of all, there is the development of an efficient and safe carrier system to deliver these compounds into the human body. Secondly, these systems will need to be optimized in order to achieve the best balance between successful delivery, intrinsic toxicity (if any), and avoidance of undesired immune responses (in the case of antisense oligos). Finally, even when all these achievements are met, there will still be the need to optimize recurrent-administration protocols to determine the uptake levels, clearance, and accumulation in various tissues (this is an often overlooked, since none of these methods will cause permanent correction of mRNA splicing defects).

Nonetheless, the first results of this exciting news strategy are available, and there is the distinct possibility that RNA-based treatment of cancer may soon enter the application stage.

## Figures and Tables

**Table 1:** Alternative splicing events detected in protein coding genes and pseudogenes implicated in cancer.

Alternative splicing event	Protein coding genes	CanGEM gene list (31057 entries)	% cancer protein coding genes
Cassette exon (CE)	13506	11771	87
Intron retention (IR)	8190	6775	83
Alternative 3′ss (A3SS)	6701	5685	85
Alternative 5′ss (A5SS)	6624	5640	85
Alternative first exons (AFE)	8050	7136	89
Alternative last exons (ALE)	3631	3233	89
Mutually exclusive exons (MXE)	3376	3006	89

Alternative splicing event	Pseudogenes	CanGEM gene LIST (31057 entries)	% cancer pseudogenes

Cassette exon (CE)	434	139	32
Intron retention (IR)	465	187	40
Alternative 3′ss (A3SS)	241	91	38
Alternative 5′ss (A5SS)	218	82	38
Alternative first exons (AFE)	91	27	30
Alternative last exons (ALE)	104	32	31
Mutually exclusive exons (MXE)	50	16	32

Homo sapiens genes (GRCh37.p10) dataset (62252 total genes) was used to filter alternative splicing events in protein coding genes (22719 entries) and pseudogenes (14775 entries) and verified their presence in the Cancer GEnome Mine database (31057 entries—CanGEM, http://www.cangem.org/).

**Table 2:** Pseudoexon insertion events directly involved in cancer pathology.

Gene name	Description	Entrez gene	Size (bp) pseudoexon	Activating mutation	Reference
*APC*	Adenomatosis polyposis coli	324	167	5′ss creation	[78]
*ATM*	Ataxia telangiectasia mutated	470	58	3′ss creation	[79]
*ATM*			65	SRE deletion	[80]
*ATM*			137	5′ss creation	[81]
*ATM*			212	5′ss creation	[82]
*BCR-ABL*	Breakpoint cluster region V-abl Abelson murine leukemia viral oncogene homolog 1 fusion	613 25	42	Genomic rearrangement	[83]
*BCR-ABL*			35	Unknown	[84, 85]
*BRCA1*	Breast cancer 1, early onset	672	66	3′ss creation	[86]
*BRCA2*	Breast cancer 2, early onset	675	93	Downstream 3′ss deletion	[87]
*BRCA2*			95	5′ss creation	[88]
*ESR1*	Estrogen receptor 1	3467	69	5′ss creation	[89]
*MSH2*	MutS homolog 2, colon cancer, nonpolyposis type 1 (*E. coli*)	4436	75	5'ss creation	[90]
*NF1*	Neurofibromin 1 (neurofibromatosis, von Recklinghausen disease, Watson disease)	4763	67/99	3′ss creation	[91]
*NF1*			70	5′ss creation	[92]
*NF1*			107	5′ss creation	[92]
*NF1*			172	3′ss creation	[93]
*NF1*			58	3′ss creation	[94]
*NF1*			76	5′ss creation	[94]
*NF1*			54	5′ss creation	[95]
*NF1*			177	5′ss creation	[92, 96, 97]
*NF2*	Neurofibromin 2 (bilateral acoustic neuroma)	4771	106	BP creation	[98]
*RB1*	Retinoblastoma 1 (including osteosarcoma)	5925	103	3′ss creation	[99]
*TSC2*	Tuberous sclerosis 2	7249	89	5′ss creation	[100]
*WRN*	Werner syndrome	7486	106	5′ss creation	[101]
*WRN*			69	3′ss creation	[102]

**Table 3:** Pseudoexons described in cancer-related genes.

Gene name	Description	Entrez gene	Size (bp) pseudoexon	Activating mutation	Reference
*ABCC8*	ATP-binding cassette, sub-family C (CFTR MRP), member 8	6833	76	5′ss creation	[103]
*ALDH7A1*	Aldehyde dehydrogenase 7 family, member A1	501	36	5′ss mutation	[104]
*CD40LG*	CD40 ligand (TNF superfamily, member 5, hyper-IgM syndrome)	959	59	5′ss creation	[105]
*CEP290*	Centrosomal protein 290 kDa	80184	128	5′ss creation	[106, 107]
*CHM*	Choroideremia (Rab escort protein 1)	1121	98	3′ss creation	[108]
*COL4A3*	Collagen, type IV, alpha 3 (Goodpasture antigen)	1285	74	3′ss creation	[109]
*COL11A1*	Collagen, type XI, alpha 1	1301	50	5′ss creation	[110]
*CTDP1*	CTD (carboxy-terminal domain, RNA polymerase II, polypeptide A) phosphatase, subunit 1	9150	95	5′ss creation	[111]
*CYBB*	Cytochrome b-245, beta polypeptide (chronic granulomatous disease)	1536	56	5′ss creation	[112]
*CYBB*			61	5′ss creation	[113]
*CYP17A1*	Cytochrome P450, family 17, subfamily A, polypeptide 1	1586	94	Upstream 5′ss mutation	[114]
*QDPR*	Quinoid dihydropteridine reductase	5860	152	5′ss creation	[115]
*DPYD*	Dihydropyrimidine dehydrogenase	1806	44	5′ss creation	[116]
*FBN1*	Fibrillin 1	2200	93	5′ss creation	[117]
*GHR*	Growth hormone receptor	2690	102	SRE deletion	[118, 119]
*GUSB*	Glucuronidase, beta	2990	68	5′ss creation	[120]
*HADH*	Hydroxyacyl-coenzyme A dehydrogenase	3033	141	5′ss creation	[103]
*HADHB*	Hydroxyacyl-coenzyme A dehydrogenase 3-ketoacyl-coenzyme A thiolase enoyl-coenzyme A hydratase (trifunctional protein), beta subunit	3032	56 106	5′ss creation	[121]
*HSPG2*	Heparan sulfate proteoglycan 2	3339	141	5′ss creation	[103]
*SMARCB1*	SWI SNF related, matrix associated, actin dependent regulator of chromatin, subfamily b, member 1	6598	72	5′ss creation	[122]
*ISCU*	Iron-sulfur cluster scaffold homolog (*E. coli*)	23479	86 100	3′ss creation	[123–125]
*SLC14A1 (JK)*	Solute carrier family 14 (urea transporter), member 1 (Kidd blood group)		136	Internal 7 kb deletion	[126]
*MCCC2*	Methylcrotonyl-coenzyme A carboxylase 2 (beta)	64087	64	SRE deletion	[127]
*MFGE8*	Milk fat globule-EGF factor 8 protein	4240	102	SRE creation	[128]
*MLC1*	Megalencephalic leukoencephalopathy with subcortical cysts 1	23209	246	5′ss creation	[129]
*MTRR*	5-methyltetrahydrofolate-homocysteine methyltransferase reductase	4552	140	SRE creation	[130]
*GPR143*	G protein-coupled receptor 143	4935	165	3′ss creation	[131]
*OAT*	Ornithine aminotransferase (gyrate atrophy)	4942	142	5′ss creation	[132]
*OFD1*	Oral-facial-digital syndrome 1	8481	62	5′ss creation	[133]
*OTC*	Ornithine carbamoyltransferase	5009	135	3′ss creation	[134]
*PCCA*	Propionyl-coenzyme A carboxylase, alpha polypeptide	5095	84	SRE creation	[135]
*PCCB*	Propionyl-coenzyme A carboxylase, beta polypeptide	5096	72	5′ss creation	[135]
*PHEX*	Phosphate regulating endopeptidase homolog, X-linked (hypophosphatemia, vitamin D resistant rickets)	5251	50 100 170	5′ss creation	[136]
*PKHD1*	Polycystic kidney and hepatic disease 1 (autosomal recessive)	5314	116	5′ss creation	[137]
*PMM2*	Phosphomannomutase 2	5373	66	3′ss creation	[138]
*PMM2*			123	5′ss creation	[138, 139]
*PRPF31*	PRP31 pre-mRNA processing factor 31 homolog (*S. cerevisiae*)	26121	175	5′ss creation	[140]
*RHD*	Rh blood group, D antigen	6007	170	Upstream 3′ss deletion and SNP in int7	[141]
*RYR1*	Ryanodine receptor 1 (skeletal)	6261	119	5′ss creation	[142]
*SLC12A3*	Solute carrier family 12 (sodium chloride transporters), member 3	6559	238	5′ss creation	[143]
*USH2A*	Usher syndrome 2A (autosomal recessive, mild)	7399	152	5′ss creation	[144]
